# A vasculogenic mimicry subtype unveiled by integrated multi-omics predicts prognosis and guides immunotherapy in MIBC

**DOI:** 10.3389/fbinf.2026.1796762

**Published:** 2026-05-29

**Authors:** Luqing Wei, Shuzhen Liao, Xin Feng, Zhenyou Tang, Zhenyu Tang, Yuting Tao, Youwen Zheng, Tao Liu, Zezu Qin, Xuancheng Cao, Zhenkun Tang, Yuxin Liu, Yiwei Cai, Tianyu Li, Chao Feng, Qiuyan Wang, Zhong Tang

**Affiliations:** 1 School of Information and Management, Guangxi Medical University, Nanning, China; 2 Center for Genomic and Personalized Medicine, Guangxi Medical University, Nanning, China; 3 Department of Urology, The First Affiliated Hospital of Guangxi Medical University, Nanning, China; 4 The Academician Workstation on Metabolism and Health, The First Affiliated Hospital of Guangxi Medical University, Nanning, China; 5 Institute of Collaborative Innovation, University of Macau, Macau, China; 6 Department of Computer Engineering, University of California, Irvine, CA, United States; 7 School of Humanities and Social Sciences, Guangxi Medical University, Nanning, China

**Keywords:** immunotherapy, molecular subtyping, muscle-invasive bladder cancer, tumor microenvironment, vasculogenic mimicry

## Abstract

**Introduction:**

The progression, recurrence, and metastasis of muscle-invasive bladder cancer (MIBC) are closely associated with vasculogenic mimicry (VM). Establishing a VM-based molecular subtyping system and clarifying its regulatory network are essential to overcome current therapeutic limitations in MIBC.

**Methods:**

This study collected 1,489 previously reported VM-related genes and identified differentially expressed genes between tumor cells and endothelial cells using single-cell transcriptomic analysis. The intersection of these gene sets yielded VM-related genes, of which 12 were selected to construct a prognostic model validated by multi-omics data, including bulk transcriptomic, genomic, mass cytometry, spatial transcriptomic, and metabolomic datasets. Single-cell and bulk RNA sequencing data were integrated to develop a VM-based molecular subtyping system.

**Results:**

MIBC patients were classified into three subtypes: VM, Mixed-VM, and Non-VM, each showing distinct prognostic outcomes consistently validated across multiple independent cohorts. The VM subtype demonstrated the most aggressive clinical behavior, characterized by high cellular plasticity and extensive metabolic reprogramming. Compared to the Non-VM subtype, VM-subtype patients exhibited an immunosuppressive tumor microenvironment and elevated tumor mutational burden (TMB), indicating a potentially favorable response to immunotherapy. Combined treatment with the MEK inhibitor TAK-733 and immune checkpoint inhibitors was proposed as a promising therapeutic strategy for this subgroup.

**Conclusion:**

This novel VM-based molecular subtyping system for MIBC shows strong potential for clinical application in prognosis prediction, immunotherapy response evaluation, and targeted drug discovery, providing a framework to guide personalized treatment strategies for MIBC patients.

## 1 Introduction

Bladder cancer ranks among the most common genitourinary malignancies and exhibits substantial molecular heterogeneity, aggressive invasiveness, and variable clinical outcomes (Dyrskjot et al., 2023). Muscle-invasive bladder cancer (MIBC) represents one of the most aggressive subtypes of bladder cancer (Patel et al., 2020). Platinum-based chemotherapy remains the standard first-line treatment, yet the median survival of patients with MIBC has shown minimal improvement over the past 3 decades (Sung et al., 2021; Powles et al., 2022). Although immune checkpoint blockade has transformed the therapeutic landscape, only a limited proportion of patients derive clinical benefit (Lenis et al., 2020). Reliable biomarkers are urgently required to achieve accurate patient stratification, improve prognostic precision, and guide individualized treatment strategies.

Vasculogenic mimicry (VM) represents a tumor vascularization mechanism that circumvents endothelial-dependent angiogenesis. Instead of endothelial cells, tumor cells interconnect and self-organize into PAS-positive, CD31-negative tubular structures, forming auxiliary channels that supplement conventional vasculature (Luo et al., 2020; Xu et al., 2021). These tumor cell-derived networks function as an alternative blood supply system distinct from traditional endothelial vessels (Xu et al., 2021). VM has been documented in various malignancies, including breast, bladder, gastric, and hepatocellular carcinomas, and is consistently linked to increased metastatic potential and poor clinical outcomes (Xu et al., 2021; Lapkina et al., 2023). In bladder cancer, VM is particularly prevalent in moderately to poorly differentiated and advanced-stage tumors, with lymph node metastasis rates reaching 75%, underscoring its critical role in invasion and disease progression (Lin et al., 2023). Despite recognition of VM in bladder tumors, its potential for precision therapy remains insufficiently explored, and no clinically applicable VM-based molecular classification has yet been established.

Rapid declines in whole-genome sequencing costs are transforming cancer diagnosis and management. Tumors once categorized by anatomical site are now divided into molecular subtypes that capture distinct transcriptional programs. These refined classifications have elucidated genotype–phenotype relationships and are increasingly incorporated into prognostic frameworks and therapeutic decision-making. A VM-based classifier for MIBC was developed, and the biological features of the VM subtype were characterized through integrated multi-omics analysis. The model accurately predicted patient outcomes, immunotherapy responsiveness, and potential therapeutic targets, providing a foundation for more precise and individualized management of MIBC.

## 2 Materials and methods

### 2.1 Data download and processing

The training cohort comprised resected tumor tissues from 52 consecutive patients with MIBC who underwent surgery at the First Affiliated Hospital of Guangxi Medical University between February 2018 and October 2019. All patients had primary, treatment-naïve tumors and had not received chemotherapy or radiotherapy before sample collection. The study was approved by the Ethics and Human Trials Committees of The First Affiliated Hospital of Guangxi Medical University (Approval of ethical number 2025-S1113), and written informed consent was obtained from all participants.

Model validation was performed across four transcriptomic layers. TCGA-MIBC RNA-seq data with matched clinical information (n = 403) served as the primary benchmark. Two GEO microarray datasets, GSE13507 (n = 165) and GSE32894 (n = 224), provided independent validation, while GSE171351 (n = 4) contributed Visium-based spatial transcriptomics. Single-cell data were retrieved from the GSA-Human database (HRA000212). To assess immunotherapy response, IMvigor210 (n = 298) was analyzed. Cross-cancer validation involved six TCGA cohorts: HNSC (n = 500), ACC (n = 79), KIRC (n = 526), LIHC (n = 334), PAAD (n = 176), and READ (n = 157), as detailed in Supplementary Table S1.

A bladder cancer–specific catalogue of VM-related genes was compiled by integrating data from GeneCards (https://www.genecards.org) and manually curated literature sources, as detailed in Supplementary Table S2. To characterize the immune landscape, bulk RNA-seq profiles were analyzed using seven deconvolution algorithms, including CIBERSORT, EPIC, xCell, ESTIMATE, quanTIseq, MCP-counter, and the Immunophenotype Score, implemented through the Immuno-Oncology Biological Research (IOBR) framework (Zeng et al., 2021). ssGSEA was then applied to generate patient-specific VM-related risk signatures, with corresponding scores provided in Supplementary Table S3. These data enabled a systematic assessment of how immune configurations vary across VM-defined risk subtypes.

### 2.2 PAS/CD31 double staining

PAS/CD31 double staining was performed on formalin-fixed paraffin-embedded (FFPE) tissue sections (4 μm) from five patients with the VM subtype and five patients with the non-VM subtype of MIBC. After deparaffinization and rehydration, heat-induced antigen retrieval was carried out in citrate buffer (pH 6.0). Endogenous peroxidase activity was blocked with 3% H_2_O_2_, followed by incubation with a rabbit monoclonal anti-CD31 antibody (Cell Signaling Technology, Danvers, MA, United States; 1:100) overnight at 4 °C. The sections were then treated with HRP-conjugated secondary antibody, and CD31 was visualized using 3,3′-diaminobenzidine (DAB). Subsequently, the same sections were subjected to periodic acid–Schiff (PAS) staining: oxidation in 0.5% periodic acid for 10 min, followed by Schiff’s reagent for 20 min at room temperature. After counterstaining with Mayer’s hematoxylin, the slides were dehydrated, cleared, and mounted with neutral resin. In VM-positive cases, CD31-negative but PAS-positive channel-like structures were identified, while true blood vessels were double positive for CD31 and PAS.

### 2.3 Single-cell RNA sequencing dataset analysis

Single-cell RNA sequencing data from eight treatment-naïve bladder cancer patients (GEO: GSE146137) (Chen et al., 2020)were analyzed. Genes detected in fewer than 3 cells and cells with fewer than 200 or more than 7,000 features, or with over 10% mitochondrial reads, were excluded. Data were log-normalized and processed using PCA and graph-based clustering in Seurat 4.4.3. Batch effects were corrected with Harmony (Korsunsky et al., 2019), followed by k-nearest neighbor graph construction and clustering with FindNeighbors and FindClusters, yielding 32 transcriptionally distinct clusters. Cell types were annotated using canonical lineage markers: epithelial tumor cells (EPCAM+, KRT8+), endothelial cells (PECAM1+, VWF+), fibroblasts (COL1A1+), myeloid cells (LYZ+, CD68^+^), T cells (CD3D+), B cells (CD79A+), and mast cells (TPSAB1+). Differential expression between tumor epithelial and endothelial compartments was identified using FindMarkers for downstream analyses.

### 2.4 Construction and validation of the VM-related prognostic risk model

Single-cell transcriptomes from bladder tumors were analyzed to isolate malignant epithelial and adjacent endothelial compartments, followed by the extraction of genes distinguishing the two. A total of 4,366 tumor-enriched transcripts were identified. Cross-referencing these with 1,489 VM-related genes from GeneCards and primary literature yielded 291 VM-associated genes specific to bladder cancer. Prognostic evaluation in the 52-patient Guangxi cohort applied univariate Cox regression (*P* < 0.05), followed by LASSO-Cox modeling using glmnet. Ten-fold cross-validation selected the optimal penalty parameter, generating a 12-gene risk model (AURKA, C1QBP, DKC1, FOXD1, MT-CO2, POMGNT2, PTN, PTPN2, SLC7A8, SOD1, TK1, TUBB) tailored to MIBC VM biology. Risk scores stratified patients into three groups: VM subtype (the upper quartile of the VM score), Non-VM subtype (the lower quartile of the VM score), and Mixed-VM subtype (interquartile range). Model performance was assessed using time-dependent ROC analysis via time ROC, and the results were validated in the TCGA and two independent GEO cohorts to confirm reproducibility and generalizability.

### 2.5 Weighted gene Co-expression network analysis

WGCNA was performed using the WGCNA package (Langfelder and Horvath, 2008) to construct a scale-free co-expression network and identify modules associated with the VM subtype. After hierarchical clustering, outlier samples that compromised network stability were removed. Soft-thresholding powers ranging from 1 to 20 were evaluated, and the power that optimized both scale-free topology fit (*R* ^2^) and mean connectivity was selected. A Pearson correlation matrix was transformed into an adjacency matrix, which was then raised to the selected power and converted into a topological overlap matrix (TOM) to capture higher-order gene interconnections. The dissimilarity matrix (1–TOM) was subjected to hierarchical clustering, and dynamic tree cutting (minimum module size = 30, deepSplit = 1) was used to identify co-expression modules. Highly similar modules were merged at a cut height of 0.25. Module–trait correlations were calculated using Pearson’s correlation, and modules with r > 0.4 and *p* < 0.05 were retained as VM-associated networks, visualized in heatmaps.

### 2.6 Spatial transcriptome data processing and analysis

Raw spatial transcriptomic data of human bladder tumors were obtained from the public repository under accession number GSE171351. Spatial spots with fewer than 100 confidently detected genes were excluded to minimize technical noise. Deconvolution of the filtered data was performed using the bladder cancer–specific signature matrix integrated into the SpaCET R package (Ru et al., 2023), yielding cell-type proportion estimates for each spot. Local activity of patient-specific bladder cancer gene modules listed in Supplementary Table S4 was quantified using the GeneSetScore algorithm. Spatial Feature mapping then transformed these enrichment scores into color gradients, enabling visualization of the spatial distribution of each gene program within the TME.

### 2.7 Metabonomics and lipidomics data analysis

Tissue collection and subsequent data acquisition followed a previously standardized metabolomic and lipidomic workflow (Feng et al., 2021a) without procedural deviation. Proportional profiles and discriminant features within each omics layer were visualized using the ggplot2 package in R to ensure transparent data presentation. Differences in metabolite and lipid abundance across samples were visualized using the pheatmap package, which condensed thousands of molecular intensity values into color-coded heatmaps that represent metabolic divergence.

### 2.8 CyTOF data analysis

CyTOF data acquisition and analysis followed a previously standardized workflow (Feng et al., 2021b) without deviation. From each tissue sample, a random subset of 5,000 cells was extracted using the cytofkit R package (Chen et al., 2016)and pooled for analysis. Ion counts for surface markers were arcsinh-transformed with a cofactor of 5 to compress extreme values while preserving relative differences. FlowSOM was used to organize the high-dimensional data into self-organizing maps, followed by PhenoGraph clustering to aggregate nodes into phenotypically coherent meta-clusters. The transformed data matrix was then projected into two dimensions using t-SNE (perplexity = 30, 2,500 iterations) for visualization. Scatter plots were generated with ggplot2, and cluster-specific marker expression was visualized as color gradients in heatmaps using the pheatmap package.

### 2.9 Mutational analysis

Masked somatic mutation files for the TCGA-BLCA cohort were obtained from the GDC Data Portal and used as the mutation matrix. Mutation types, variant allele frequencies, and sample-level distributions were analyzed using the maftools R package (Mayakonda et al., 2018) to generate an oncoprint summarizing the mutational landscape. TMB was derived from whole-exome sequencing data by counting all non-synonymous single-nucleotide variants and small insertions or deletions per sample using dplyr. The counts were normalized to 38 Mb, the standard exome size, to yield mutations per megabase, in accordance with international TMB harmonization guidelines.

### 2.10 Drug sensitivity analysis

Drug sensitivity analysis was conducted to identify compounds with selective activity against VM-positive tumors. Data were obtained from the Genomics of Drug Sensitivity in Cancer (GDSC) database (cancerRxgene.org) (Yang et al., 2013), the largest publicly available repository linking drug response to genomic features. The oncoPredict algorithm (Maeser et al., 2021)was used to project bulk RNA-seq profiles onto pre-trained pharmacogenomic models. Samples were divided into VM-positive and VM-negative groups, and genome-wide differential expression profiles were used to estimate 198 half-maximal inhibitory concentrations (IC_50_) for each drug. Compounds showing IC_50_ differences exceeding one log-fold between subtypes were retained as candidates with subtype-specific sensitivity. The VM gene signature was further queried against the Connectivity Map (CMap, Broad Institute) (Lamb et al., 2006)to identify small molecules whose transcriptional effects inversely correlated with the VM program, thereby highlighting potential repositioned therapeutics for MIBC.

### 2.11 Molecular docking analysis

Molecular docking analysis was performed using the CB-DOCK2 platform (Liu et al., 2022). Three-dimensional structures of candidate compounds were retrieved from PubChem, and corresponding target protein structures were obtained from the Protein Data Bank (PDB). Ligand and receptor coordinates were uploaded to CB-DOCK2 for automated docking without preprocessing. The server explored rotational and translational conformations, scored each pose, and identified the minimum-energy configuration. Binding affinity, expressed as negative kcal·mol^-1^, served as the sole ranking criterion, with lower energy indicating stronger predicted binding. Complexes with the most favorable docking scores were visualized in molecular graphics software to examine hydrogen bonding, π–π interactions, and salt bridges that stabilize ligand–receptor interactions.

### 2.12 Statistical analysis

All statistical analyses were conducted using R version 4.4.3 and GraphPad Prism version 10.1.2. Comparisons between VM and Non-VM tumors employed either Student’s t-test or the Wilcoxon rank-sum test, depending on data distribution and variance homogeneity; nonparametric tests were applied when skewness or heteroscedasticity was present. To account for multiple comparisons, the Benjamini, Krieger, and Yekutieli (BKY) two-stage step-up method was used to control the False Discovery Rate (FDR). Survival analyses were performed using Kaplan–Meier estimation, with group differences assessed by the log-rank test. Correlations between variables were evaluated using Pearson’s correlation coefficient. In these multi-comparison analyses, an adjusted P-value (q-value) less than 0.05 (corresponding to an FDR of 5%) was considered statistically significant.

## 3 Results

### 3.1 Identification of signature genes defining the VM subtype in MIBC

**FIGURE 1:**
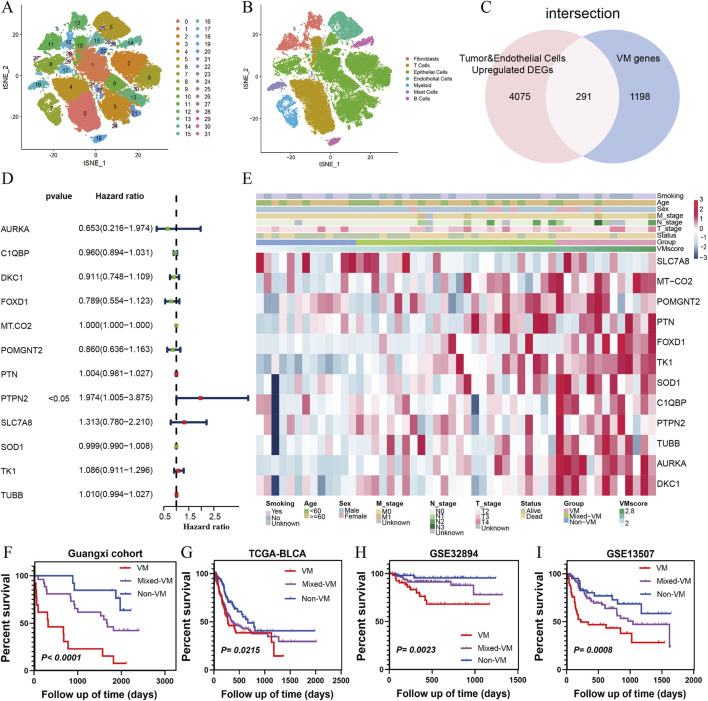
Identification of signature genes for vasculogenic mimicry (VM) subtypes in MIBC. **(A)** t-SNE visualization showing 32 cell clusters identified by unsupervised clustering of scRNA-seq data from 8 bladder cancer samples. **(B)** Annotation of 7 major cell types based on the expression of classic marker genes for the clusters shown in **(A)**. **(C)** Selection of VM-associated candidate genes by intersecting upregulated differentially expressed genes in tumor cells with a known VM-related gene set. **(D)** Forest plot of the multivariate Cox regression model used to construct a VM-related prognostic signature, comprising 12 genes. **(E)** Heatmap displaying the association between clinicopathological features and the expression patterns of the 12-gene VM signature in the Guangxi cohort. **(F)** Kaplan-Meier curves for overall survival (OS) of patients stratified into three VM subtypes based on the prognostic signature in the Guangxi cohort. **(G–I)** Kaplan-Meier survival analysis validating the OS of the three VM subtypes in the TCGA-BLCA **(G)**, GSE32894 **(H)**, and GSE13507 **(I)** cohorts, respectively.

To establish a VM-centered prognostic framework for MIBC and to characterize the heterogeneity and therapeutic vulnerabilities of VM-enriched subgroups, single-cell RNA sequencing data from eight freshly resected tumors were analyzed. Unsupervised clustering partitioned the transcriptomic landscape into 32 distinct clusters (Figure 1A). Manual annotation classified these clusters into seven canonical cell lineages (fibroblasts, T cells, epithelial cells, endothelial cells, myeloid cells, mast cells, and B cells), thereby constructing a comprehensive cellular atlas (Figure 1B). Because VM channels originate from highly plastic tumor cells that transiently adopt vasculogenic features rather than from endothelial proliferation, malignant epithelial and endothelial populations were extracted for differential expression analysis. Intersection of the 4,366 genes upregulated in tumor cells with a curated panel of 1,489 VM-related genes derived from published studies and public databases identified 291 VM-associated genes specific to bladder cancer (Figure 1C). These 291 candidates underwent univariate Cox regression, least absolute shrinkage and selection operator (LASSO) regularization, and multivariate Cox refinement, resulting in a 12-gene prognostic signature comprising AURKA, C1QBP, DKC1, FOXD1, MT-CO2, POMGNT2, PTN, PTPN2, SLC7A8, SOD1, TK1, and TUBB (Figure 1D). The combined expression profile of these genes effectively stratified patient outcomes. Independent validation using the Human Protein Atlas confirmed that AURKA, C1QBP, DKC1, POMGNT2, PTN, SLC7A8, SOD1, and TK1 exhibited markedly higher expression levels in tumor tissues compared with matched normal urothelium (Supplementary Figure S1).

VM score potential was quantified across 52 Guangxi MIBC samples using the ssGSEA algorithm applied to bulk transcriptomic data. The resulting VM score distribution divided the cohort into three biologically distinct groups: VM subtype(the upper quartile of the VM score),Non-VM subtype (the lower quartile of the VM score), and an intermediate Mixed-VM subtype. VM subtype tumors exhibited more aggressive clinicopathological characteristics, including higher grade and advanced stage, and were associated with the poorest survival outcomes. Mixed-VM subtype cases showed intermediate prognoses, whereas Non-VM subtype patients displayed the most favorable disease course (Figure 1E,F). The same stratification pattern reproduced comparable survival trends in the TCGA-BLCA, GSE32894, and GSE13507 cohorts (Figures 1G–I). Both univariate and multivariate analyses confirmed the VM score as an independent prognostic factor (Supplementary Figure S2). In addition, PAS/CD31 double staining results confirmed that more typical VM structures were observed in VM subtype tumors than Non-VM subtype tumors, suggesting that this subtyping system was reliable (Supplementary Figure S3).

### 3.2 Different VM subtypes exhibit distinct transcriptomic signatures

**FIGURE 2:**
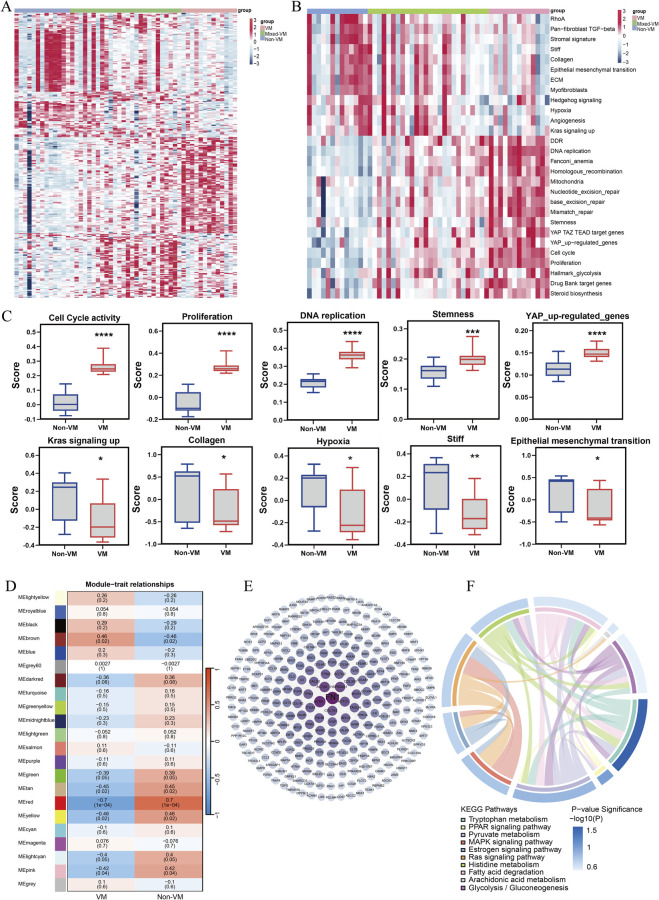
Three vasculogenic mimicry (VM) subtypes exhibit distinct transcriptomic characteristics. **(A)** Heatmap displaying the top 100 most significantly differentially expressed genes among the three VM subtypes. **(B)** Heatmap comparing the activity levels of the three VM subtypes based on ssGSEA scores for specific gene sets. **(C)** Box plots comparing the enrichment scores of specific signature gene sets between VM and Non-VM subtypes. * indicates q < 0.05; ** indicates q < 0.01; *** indicates q < 0.001; **** indicates q < 0.0001. **(D)** Key co-expression modules significantly associated with VM subtypes, as identified by weighted gene co-expression network analysis (WGCNA). **(E)** Protein-protein interaction (PPI) network analysis identifying hub genes within the brown module shown in **(D)**. **(F)** KEGG pathway enrichment analysis of genes in the brown module identified in **(D)**.

To explore the molecular framework of the VM subtype, the entire transcriptome was examined for differential expression patterns. Among the three VM-defined subtypes, the transcriptional landscape displayed sharply divergent profiles (Figure 2A). A curated malignancy-progression signature was then projected onto the Guangxi bladder cancer cohort, revealing a pronounced polarization (Figure 2B). The VM subtype showed enrichment of stemness, proliferation, cell cycle, and YAP pathway effectors. In contrast, the Non-VM group maintained a stromal-dominant phenotype, characterized by elevated expression of extracellular matrix and collagen-related genes (Figure 2C). Weighted gene co-expression network analysis (WGCNA) identified the brown module, whose expression pattern closely paralleled the VM subtype (Figure 2D). Functional enrichment analysis linked this module to the MAPK pathways and tumor metabolic pathways (Figure 2F). Protein-protein interaction (PPI) mapping highlighted fibronectin-1 (FN1) as the central hub within this module, suggesting FN1 was significantly associated with the invasiveness of the VM subtype and served as a potential regulatory candidate (Figure 2E). Previous studies confirmed in other solid tumor models that hub genes, including *FN1, ACTA2*, and *COL1A1*, participated in extracellular matrix remodeling and promoted endothelial-like phenotypic transitions(Liu et al., 2025; Zheng et al., 2025), providing a biological rationale that supported our computational results.

**FIGURE 3:**
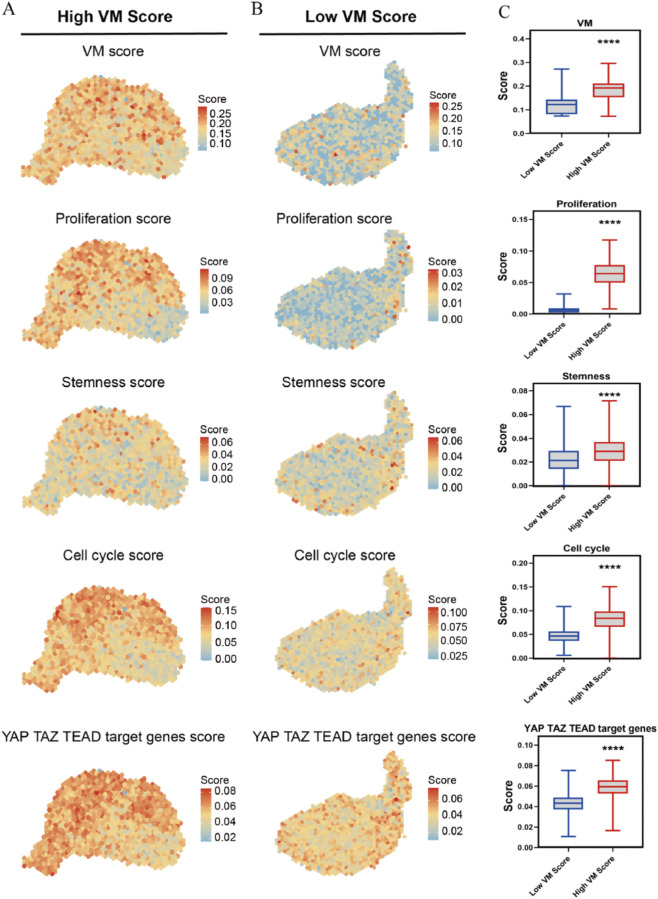
Spatial transcriptomic analysis reveals transcriptomic features in patients with different VM scores. **(A,B)** Spatial plots display the differences in signature scores between patients with high VM scores and those with low VM scores. **(C)** Box plots show the differences in VM signature, proliferation signature, stemness signature, cell cycle signature, and YAP signaling pathway activation scores between patients with high and low VM scores.* indicates q < 0.05; ** indicates q < 0.01; *** indicates q < 0.001; **** indicates q < 0.0001.

Spatial transcriptomic analysis further clarified the topographic organization of VM-associated programs. Expression mapping of the twelve core VM genes across bladder cancer tissue generated a VM score for each spatial spot. High-scoring regions exhibited pronounced expression of cell cycle markers, enhanced stemness, and YAP signatures, mirroring the bulk RNA-seq findings (Figures 3A,B). The spatial data indicated that VM-associated programs are not diffusely distributed but rather confined to distinct, self-reinforcing niches defined by sustained self-renewal, rapid mitotic activity,and persistent YAP activation (Figure 3C). Each feature likely contributes to the aggressive progression observed in patients with the VM subtype.

### 3.3 VM subtypes display distinct patterns of metabolic reprogramming

Metabolic rewiring serves as a central mechanism governing tumor plasticity and the course of malignant progression. To define the metabolic features distinguishing VM subtypes, each subgroup was analyzed using ssGSEA with curated gene signatures encompassing major cancer-related metabolic pathways. The resulting profile revealed three distinct metabolic identities (Supplementary Figure S4A). Compared with the Non-VM subtype, the VM subtype showed marked upregulation of pyrimidine biosynthesis, steroid biogenesis, vitamin B6 metabolism, glyoxylate–dicarboxylate shunting, and folate-mediated one-carbon metabolism. In contrast, Non-VM tumors displayed enrichment of inositol phosphate turnover, β-alanine catabolism, phenylalanine metabolism, taurine/hypotaurine conversion, and arachidonate oxidation (Supplementary Figure S4B). Gene set enrichment analysis (GSEA) further expanded these findings, identifying enhanced activation of pyrimidine metabolism, N-glycan biosynthesis, enterocyte cholesterol metabolism, one-carbon metabolism, disorders of folate metabolism and transport, tRNA threonylcarbamoyladenosine metabolic process pathways in VM-enriched tissues (Supplementary Figure S4C). Collectively, these data frame the VM subtype may as a metabolically reprogrammed entity whose distinct pathway architecture may facilitate and sustain vasculogenic mimicry activity.

### 3.4 Distinct metabolite profiles characterize VM subtypes

**FIGURE 4:**
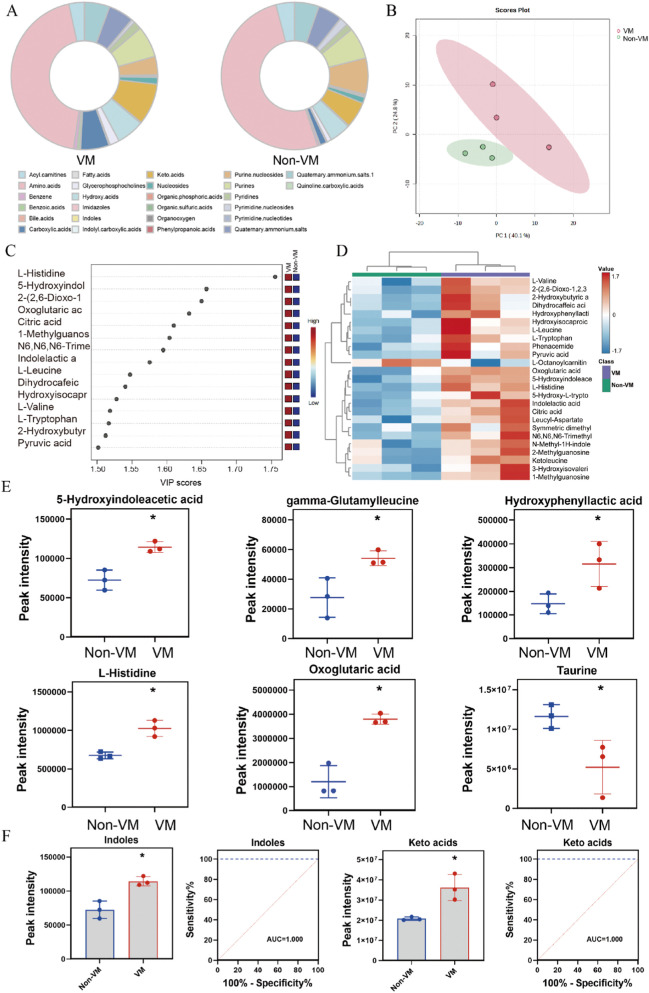
Potential metabolic biomarkers distinguishing VM and Non-VM subtypes. **(A)** Relative abundance of metabolites in VM and Non-VM subtypes. **(B)** Principal component analysis (PCA) shows a clear separation between VM and Non-VM subtypes based on their metabolic profiles. **(C)** Variable importance in projection (VIP) scores of the altered metabolites. **(D)** Heatmap of the top 25 differentially abundant metabolites in VM and Non-VM subtypes. **(E)** Peak intensities of the top 6 significantly different metabolites between VM and Non-VM subtypes. **(F)** Abundance levels and AUC values of the metabolites indoles and keto acids. * indicates q < 0.05; ** indicates q < 0.01; *** indicates q < 0.001; **** indicates q < 0.0001.

To elucidate the metabolic reprogramming underlying the VM subtype and identify small-molecule drivers of its aggressive phenotype, triplicate specimens from both VM and Non-VM tumors were analyzed using untargeted metabolomics and lipidomics. The results revealed significant differences in metabolite profiles between the two subtypes (Figure 4A). Subsequently, unsupervised principal component analysis (PCA) was applied to reduce the dimensionality of the standardized high-dimensional metabolite data, using singular value decomposition to extract the primary directions of variation. A scatter plot visualizing sample distribution based on the first two principal components revealed a clear separation trend exist between VM and Non-VM samples, indicating that their metabolic phenotypes not only exhibit statistically significant differences but are also biologically distinct (Figure 4B). Metabolite importance was assessed using variable importance in projection (VIP) scores. The fifteen metabolites with the highest VIP values emerged as key molecular determinants capable of distinguishing VM from Non-VM samples (Figure 4C). The twenty-five metabolites showing the largest differences are displayed in the heat map (Figure 4D). From these, six metabolites exhibited highly significant concentration shifts: 5-hydroxyindole-3-acetic acid, γ-glutamylleucine, 3-(4-hydroxyphenyl) lactic acid, L-histidine, 2-oxoglutaric acid, and taurine (Figure 4E). Receiver operating characteristic (ROC) analysis demonstrated that elevated indole and keto acid levels in VM lesions produced area under the curve (AUC) values approaching unity, indicating that these metabolite classes provide near-perfect discriminatory accuracy for distinguishing VM from Non-VM subtypes (Figure 4F).

### 3.5 Distinct lipid profiles define VM subtypes

**FIGURE 5:**
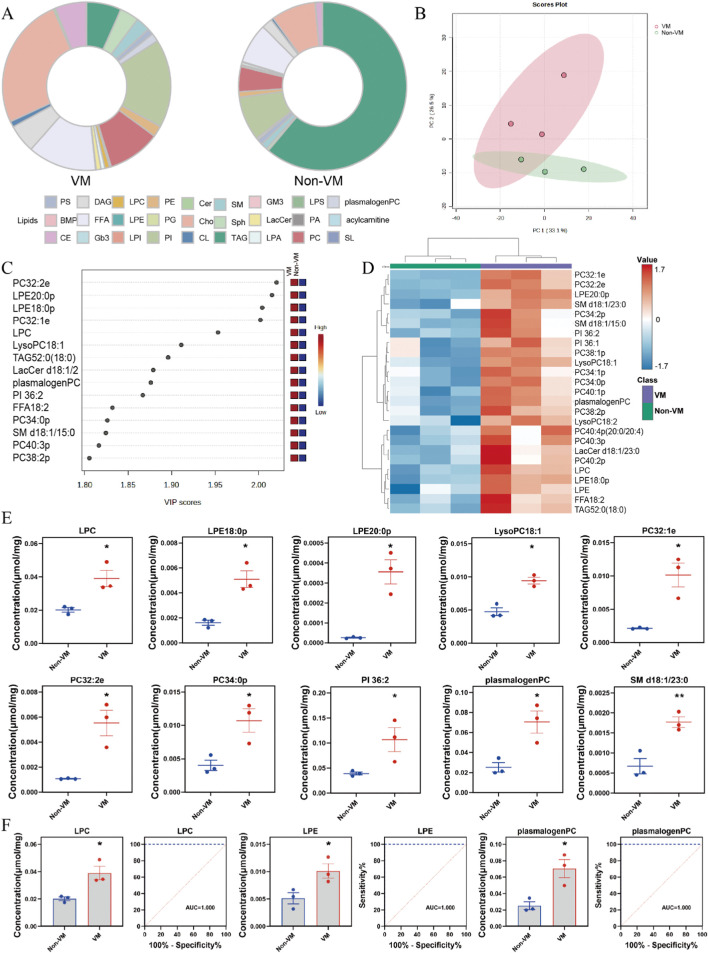
Potential lipid biomarkers distinguishing VM and Non-VM subtypes. **(A)** Relative abundance of lipids in VM and Non-VM subtypes. **(B)** Principal component analysis (PCA) showing distinct separation between VM and Non-VM subtypes based on lipid profiles. **(C)** Variable importance in projection (VIP) scores of the altered lipids. **(D)** Heatmap of the top 25 differentially abundant lipids in VM and Non-VM subtypes. **(E)** Peak intensities of the top 10 significantly different lipids between VM and Non-VM subtypes. **(F)** Concentration differences and AUC values of the lipids LPC, LPE, and plasmalogenPC. * indicates q < 0.05; ** indicates q < 0.01; *** indicates q < 0.001; **** indicates q < 0.0001.

To characterize the lipidomic differences distinguishing VM from Non-VM phenotypes, a total of 417 lipid species were profiled. The initial overview already revealed pronounced inter-subtype heterogeneity (Figure 5A). PCA further confirmed a clear separation between the two groups, demonstrating that distinct lipid expression patterns between VM and Non-VM tumors (Figure 5B). VIP analysis identified 15 key lipid species that contributed most strongly to subtype discrimination (Figure 5C). Subsequent univariate testing highlighted LPE 18:0p, LPE 20:0p, LysoPC 18:1, and SM d18:1/23:0 as the most prominent lipids enriched in VM tissues (Figures 5D,E). When analyzed at the lipid-class level, LPC, LPE, and plasmalogen-PC consistently exhibited higher abundance in the VM subtype, a pattern that ROC analysis confirmed as having strong discriminatory performance (Figure 5F). Collectively, these findings identify LPC, LPE, and plasmalogen-PC perhaps as a concise lipid signature that reliably distinguishes the VM subtype from its Non-VM counterpart.

### 3.6 The immune microenvironment is heterogeneous across VM subtypes

The initiation and persistence of VM are shaped by dynamic interactions within the tumor microenvironment (TME), which orchestrates a complex network of multicellular communication. To determine how the TME influences VM subtype formation, bulk transcriptomes were analyzed using seven complementary immune deconvolution algorithms, each capturing distinct dimensions of immune composition and activity (Supplementary Figure S5A). The integrated results revealed a strong positive association between VM scores and the relative abundance of resting macrophages, megakaryocyte–erythroid progenitors, monocytes, sebaceous gland cells, γδ T cells, and Th1 cells, indicating that the microenvironment possibly shapes VM identity (Supplementary Figure S5B).

**FIGURE 6:**
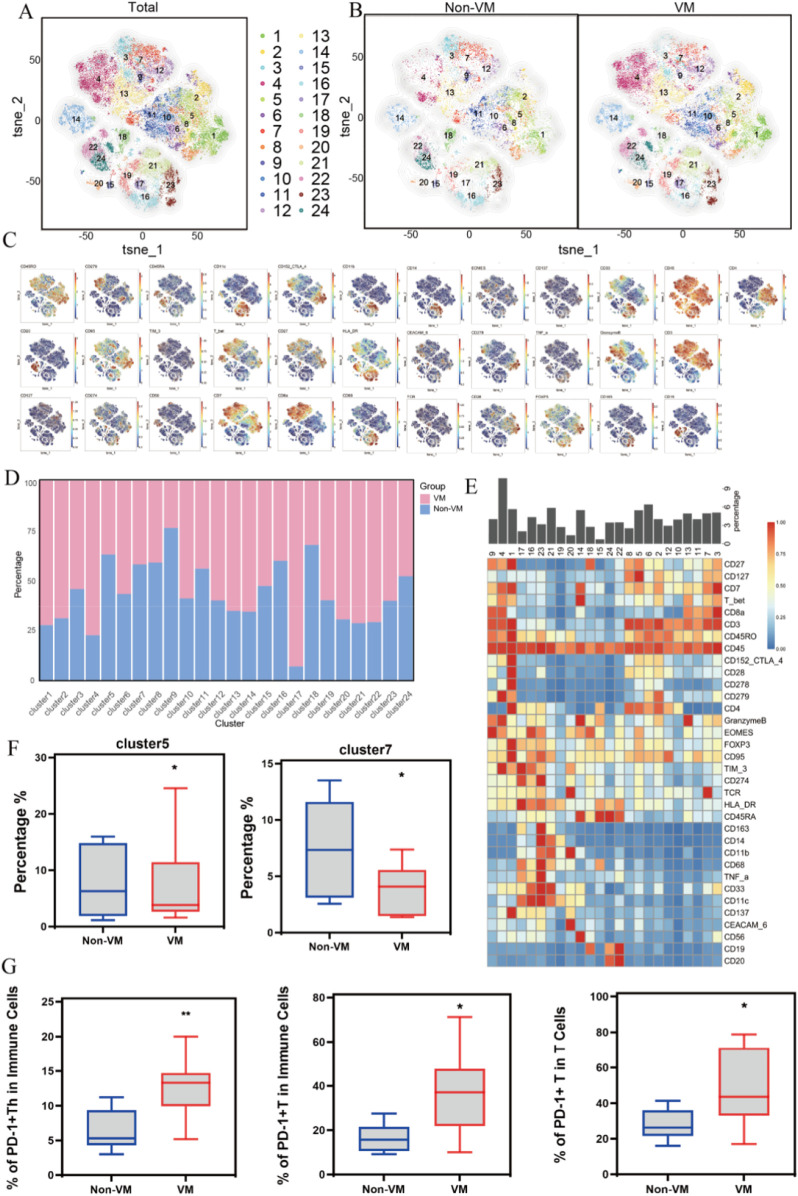
Single-cell mass cytometry reveals heterogeneity in the tumor immune microenvironment between VM and Non-VM subtypes. **(A,B)** t-SNE visualization showing all single cells from the entire cohort **(A)** and stratified by VM and Non-VM subtypes **(B)**, colored by the 24 cell clusters identified through PhenoGraph unsupervised clustering. **(C)** t-SNE projection of all single cells, colored by expression of classic cell surface markers. **(D)** Relative abundance distribution of each cell cluster in VM versus Non-VM subtypes. **(E)** Heatmap displaying the expression levels of classic markers across the 24 cell clusters. **(F)** Differences in the cellular proportions of Cluster 5 and Cluster 7 between VM and Non-VM subtypes. **(G)** Box plots comparing the proportions of PD-1+ T cells and PD-1+ Th cells among total immune cells, and PD-1+ T cells among total T cells between VM and Non-VM subtypes.* indicates q < 0.05; ** indicates q < 0.01; *** indicates q < 0.001; **** indicates q < 0.0001.

To gain higher spatial and cellular resolution, bulk-level findings were complemented by single-cell mass cytometry. Nineteen resected tumors, including 11 VM subtype tumors and 8 Non-VM subtype tumors, were dissociated and profiled using a 34-marker mass cytometry panel designed to capture immune lineage and activation states. After data normalization, t-distributed stochastic neighbor embedding (t-SNE) and PhenoGraph clustering segregated immune cells into 24 clusters, with clear compositional divergence between VM and Non-VM tumors (Figures 6A–D). Non-VM subtypes were enriched for two CD4^+^ T-cell clusters (5) and one CD8^+^ T-cell cluster (7), all characterized by low PD-1 expression. In contrast, VM tumors displayed marked accumulation of PD-1^+^ CD4^+^ and PD-1^+^ CD8^+^ T cells, indicative of an immunosuppressed T-cell compartment (Figures 6E–G). These findings suggest that VM tumors are potentially characterized by an immune milieu predisposed to T-cell dysfunction and checkpoint pathway activation, revealing an exploitable susceptibility to immune checkpoint inhibition not observed in Non-VM counterparts.

### 3.7 Identification of mutation landscape and tumor neoantigens of VM subtypes in MIBC

To characterize the genomic architecture of VM subtype tumors, the TCGA-BLCA dataset was analyzed. Missense mutations dominated the mutational spectrum, with single-nucleotide substitutions comprising the majority of events (Supplementary Figure S6B–D). Somatic alterations were detected in 188 of 198 samples (94.95%). TP53 emerged as the most frequently mutated gene, affecting 46% of tumors, while nineteen additional driver genes exhibited recurrent mutations at frequencies ranging from 12% to 41% (Supplementary Figure S6A). Comparative analysis between subtypes revealed that TP53, RB1, and ADCY2 carried significantly higher mutation burdens in the VM subtype, whereas FGFR3 alterations were more prevalent in the Non-VM subtype (Supplementary Figure S6G). TMB was also markedly elevated in the VM subtype (Supplementary Figure S6E). Survival modeling demonstrated a pronounced interaction between VM status and TMB. Among TMB-high cases, Non-VM subtype patients achieved the longest overall survival, while VM subtype patients with low TMB had the poorest outcomes. Even within identical TMB strata, Non-VM subtype individuals consistently outlived their VM subtype counterparts (Supplementary Figure S6F). Therefore, integration of VM classification with TMB status might enhance prognostic precision in MIBC.

### 3.8 Estimation of drug sensitivity in VM subtypes

To identify compounds with potential activity against the VM subtype, transcriptomic profiles were analyzed using a drug sensitivity prediction framework. The ten agents whose expression signatures most strongly anticorrelated with the VM transcriptional program are shown in Supplementary Figure S7A,B. TAK-733 ranked first, with a correlation score of −0.9181, indicating a strong inverse relationship between VM gene expression and the compound’s cytostatic effect. These findings highlight TAK-733 probably as a promising precision therapeutic candidate for VM-positive tumors. Next, the subtype-specific efficacy of conventional chemotherapeutic and targeted agents was evaluated. Estimated IC_50_ values inferred from MIBC cohorts revealed that ZM447439, GSK269962A, and Alpelisib, along with six additional compounds, exhibited lower half-maximal inhibitory concentrations in VM tumors, which may suggest enhanced sensitivity (Supplementary Figure S7C). In contrast, Entospletinib and Nutlin-3a showed right-shifted IC_50_ distributions, implying reduced efficacy in the VM subtype (Supplementary Figure S8).

These findings indicate that the first group of agents aligns with VM-associated molecular characteristics and may hold greater therapeutic promise. In silico molecular docking likely further supported these results by positioning TAK-733 within the catalytic domains of all twelve genes comprising the VM signature (Supplementary Figure S9). The comprehensive binding profile reinforces the transcriptomic predictions and provides a mechanistic justification for considering TAK-733, a MEK inhibitor, presumably as a targeted therapeutic strategy for VM-positive MIBC.

### 3.9 VM subtype associates with poor prognosis of tumors and may benefit from immunotherapy

**FIGURE 7:**
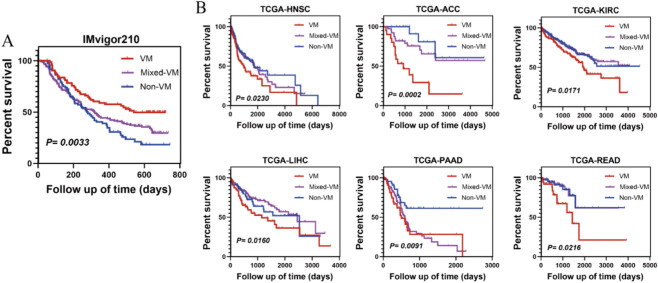
Association of VM subtypes with adverse prognosis in pan-cancer cohorts and benefit from immunotherapy. **(A)** In the IMvigor210 cohort receiving anti-PD-L1 immunotherapy, patients with the VM subtype demonstrated the most favorable overall survival. **(B)** Analysis of multiple cancer types from the TCGA pan-cancer cohort revealed that the VM subtype was significantly associated with poorer overall survival in head and neck squamous cell carcinoma (HNSC), adrenocortical carcinoma (ACC), kidney renal clear cell carcinoma (KIRC), hepatocellular carcinoma (LIHC), pancreatic adenocarcinoma (PAAD), and rectal adenocarcinoma (READ).

The collective evidence may indicate that the VM-defined subtype maintains a profoundly immunosuppressed microenvironment while harboring a high TMB, a combination that positions it as a potential responder to immune checkpoint inhibition. To evaluate whether this molecular profile predicts clinical benefit, patients from the IMvigor210 immunotherapy trial were stratified into the three VM subtypes. Survival curves showed immediate separation: patients within the VM subtype demonstrated significantly longer overall survival compared with those in the Mixed-VM or Non-VM subtypes, establishing a strong association between the VM signature and enhanced responsiveness to checkpoint blockade (Figure 7A). We further incorporated clinical information, including VM score, ECOG performance status, TMB, PD-L1 expression, gender, tumor stage, prior platinum-based/BCG therapy, and smoking history, into univariate and multivariate Cox regression analyses within the IMvigor210 cohort. The results indicated that the VM score remained a significant independent predictor for longer overall survival (HR = 0.14, 95% CI: 0.03–0.67, *P = 0.014*), even after adjusting for key confounders like ECOG status and TMB (Supplementary Figure S10). In addition, across multiple additional cancer types, including head and neck squamous cell carcinoma (HNSC), adrenocortical carcinoma (ACC), kidney renal clear cell carcinoma (KIRC), hepatocellular carcinoma (LIHC), pancreatic cancer (PAAD), and rectal cancer (READ), the VM subtype consistently correlated with poorer survival outcomes, confirming that the VM-based classification framework probably maintains prognostic relevance across diverse malignancies (Figure 7B).

## 4 Discussion

A molecular subtyping framework for MIBC was established and validated by integrating VM features with multi-omics data. The model stratifies patients into three categories (VM, Mixed-VM, and Non-VM), with the VM group consistently exhibiting the poorest survival outcomes. By transforming VM from a qualitative indicator of unfavorable prognosis into a quantitative and clinically applicable classifier, the framework probably provided a practical tool for patient stratification in clinical settings. Multi-omics profiling further refined the characterization of the VM subtype, elucidating the molecular mechanisms underlying its aggressive behavior and identifying potential therapeutic vulnerabilities.

Bulk RNA sequencing and spatial transcriptomics revealed that the VM subtype activated a cluster of pathways associated with aggressive tumor progression, including stemness, proliferation, cell-cycle regulation, and YAP signaling. The pathway profile aligned with findings from melanoma and hepatocellular carcinoma, where VM-competent cells exhibit dedifferentiation and stem-like characteristics (Hendrix et al., 2003; Zheng et al., 2021). These parallels may provide a mechanistic explanation for the heightened invasiveness and metastatic potential observed in the VM subtype. Transcriptomic analyses also indicated extensive metabolic reprogramming, characterized by upregulation of pyrimidine and purine metabolism, steroid biosynthesis, mitochondrial DNA processing, and fatty acid pathways. Metabolomic profiling further confirmed a pronounced metabolic divergence between VM and Non-VM tumors, highlighting a fundamentally restructured metabolic state as a central feature of the VM subtype. Formation of VM channels imposes substantial energetic demands. Elevated levels of 5-hydroxyindoleacetic acid, γ-glutamylleucine, L-histidine, and 2-oxoglutaric acid were detected—metabolites that fulfill both the anabolic requirements of rapidly proliferating cells and the structural needs of nascent vascular channels. Previous studies have linked 5-hydroxyindoleacetic acid and γ-glutamylleucine to enhanced tumor aggressiveness (Hanson and Serin, 1955; Ewang-Emukowhate et al., 2023; Shi et al., 2025), supporting the view that the VM-associated metabolic signature functions potentially as an active driver rather than a secondary consequence of the phenotype. Targeting these VM-specific metabolic vulnerabilities may represent a promising therapeutic strategy.

TME plays a critical role in supporting tumor cell survival and promoting malignant progression. Using CyTOF, immune cell compositions within the TME were compared between VM and Non-VM tumors at single-cell resolution. A marked enrichment of PD-1^+^CD4^+^ and PD-1^+^CD8^+^ T cells was observed in VM tumors, probably indicating the presence of a profoundly immunosuppressed niche. Genomic profiling further revealed that VM tumors possess a higher TMB, a feature that would typically increase neoantigen formation and stimulate immune activation. However, the VM-associated TME remained overtly immunosuppressive, likely due to the accumulation of PD-1^+^ T-cell clusters that counteract the immunogenicity potential of high TMB. This finding underscores why TMB alone serves as an incomplete predictor of immunotherapy efficacy. Incorporating VM status, reflecting intrinsic tumor aggressiveness, identifies a “high-TMB but immunosuppressed” subtype that exhibits limited therapeutic benefit, potentially adding a new dimension to precision immunotherapy screening. Independent immunotherapy cohorts confirmed VM subtyping potentially as a robust biomarker for predicting therapeutic response in MIBC. Patients with VM tumors may benefit from treatment combinations that integrate immune checkpoint blockade with agents targeting VM-associated pathways. Computational screening identified the MEK inhibitor TAK-733 as a potential VM-directed therapy that suppresses MAPK signaling (Jasek-Gajda et al., 2018), consistent with previous evidence linking VM activity to tumor plasticity-related pathways, including MAPK (Roesch, 2015; Villarreal et al., 2025). Published *in vitro* studies further corroborated the predicted sensitivity of MIBC cell lines to TAK-733. Specifically, it was established that “Basal-like” bladder cancer cells—which showed high molecular overlap with the VM phenotype—exhibited significantly higher sensitivity to TAK-733 compared to the Luminal subtype (Merrill et al., 2020). Additionally, evidence indicated that MEK inhibitors (such as AZD6244 and U0126) not only directly suppressed the growth of *HRAS*-mutated bladder cancer cells but also induced apoptosis via MAPK/ERK signaling blockade and enhanced the cytotoxic effects of *Bacillus* Calmette-Guérin (BCG) on cells like T24. These findings reinforced the biological rationale for targeting the MEK pathway—the primary target of TAK-733—to suppress MIBC progression (Kiessling et al., 2015; Whang et al., 2017). However, the proposed precision strategy offers a promising therapeutic avenue for patients within the poorest-prognosis VM subgroup and now requires experimental validation.

We have developed a novel VM-based molecular subtyping system for MIBC, utilizing a quadrant-based stratification, successfully identified distinct VM subtypes, demonstrating robust prognostic predictive capabilities across multiple cohorts. Nevertheless, we acknowledge that the intermediate ‘Mixed-VM’ subtype may represent a heterogeneous population in a transitional state along the VM activity spectrum, exhibiting mixed characteristics of both VM and Non-VM. This inherently introduces a degree of uncertainty in precisely stratifying these patients. Despite this, the current methodology effectively highlights the extreme phenotypes at both ends of the VM scoring spectrum. Future research will focus on optimizing this classification system, exploring more refined data-driven clustering algorithms or optimal cutoff point analyses, to enhance classification precision and cross-platform reproducibility, and to thoroughly elucidate the biological mechanisms underlying the Mixed-VM subtype. In addition, the causal mechanisms underlying VM formation—particularly how metabolic reprogramming drives this process—require further validation through *in vitro* and *in vivo* experiments. In summary, our study not only provides a new tool for precision stratification of bladder cancer but, more importantly, highlights a critical direction for future research: targeting the VM subtype with combination immunotherapies to counteract both immunosuppression and VM-mediated tumor progression. Ultimately, we anticipate that this subtyping system may be integrated into clinical decision-making to improve outcomes for MIBC patients, particularly those with the most aggressive VM subtype.

## Data Availability

The original contributions presented in the study are included in the article/Supplementary Material, further inquiries can be directed to the corresponding authors.
