# Supplementary material for: A vasculogenic mimicry subtype unveiled by integrated multi-omics predicts prognosis and guides immunotherapy in MIBC
Source: Front Bioinform. 2026 May 29;6:1796762. doi: 10.3389/fbinf.2026.1796762 (PMC13260346; doi:10.3389/fbinf.2026.1796762)
Supplement: Supplementary file 1 [file Supplementaryfile1.docx]

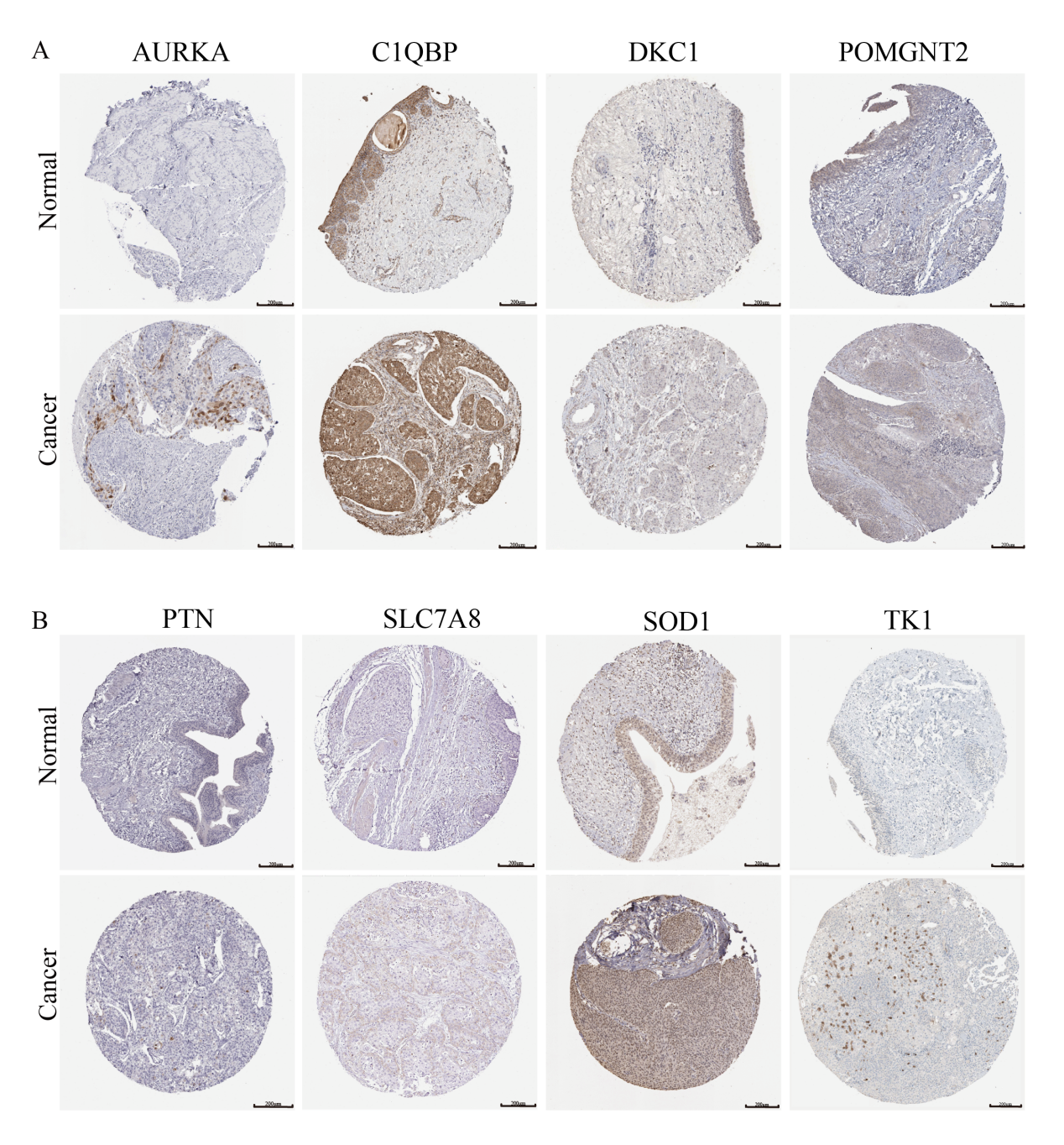


**Figure S1** Immunohistochemical validation of the expression characteristics of eight core proteins from the VM prognostic model in bladder cancer tissues. **(A-B)** Representative IHC images confirm the significant overexpression of proteins AURKA, C1QBP, DKC1, POMGNT2, PTN, SLC7A8, SOD1, and TK1 in bladder cancer tissues compared to their matched adjacent normal tissues. Scale bar: [200μm].


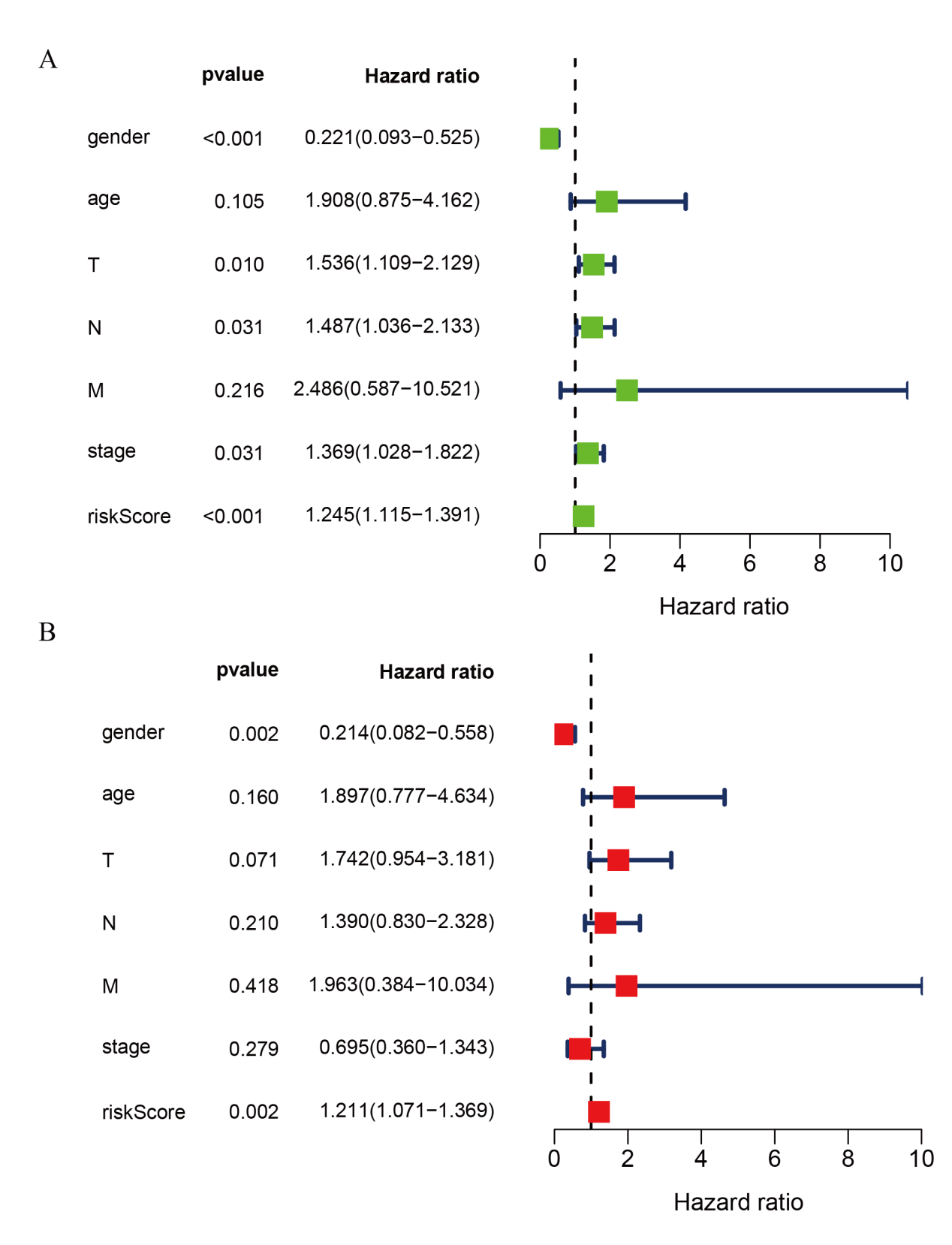


**Figure S2** Univariate and multivariate Cox regression analyses assessing the prognostic value of the VM-derived risk score. **(A)** Forest plot showing the results of univariate Cox regression analysis for overall survival. **(B)** Forest plot showing the results of multivariate Cox regression analysis for overall survival. Hazard ratios (HR) with 95% confidence intervals are shown for each clinical variable and the VM-derived risk score.


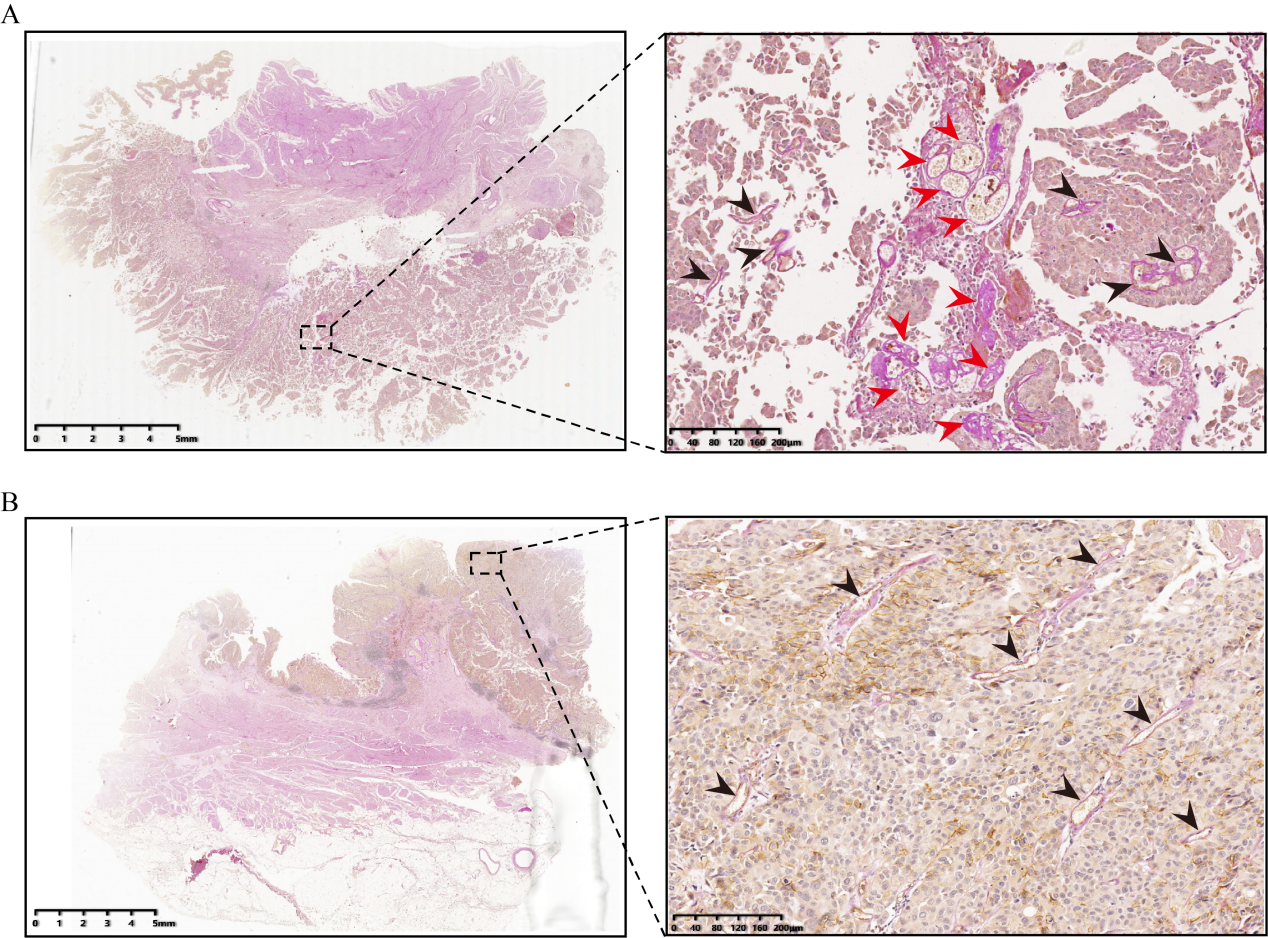


**Figure S3** Representative pathological images of vascular structures in VM subtypes and Non-VM subtypes. **(A)** Representative image of PAS/CD31 double staining in VM subtype tumor tissue. **(B)**Representative image of PAS/CD31 double staining in Non-VM subtype tumor tissue..The whole slide scan is on the left, and the area within the dotted box is shown enlarged on the right. The red arrow indicates the vascular mimetic lumen (PAS+/CD31-); the black arrow indicates the traditional endothelial microvessel (PAS+/CD31+). The left scale bar = 5 mm; the enlarged right scale bar = 200 μm.


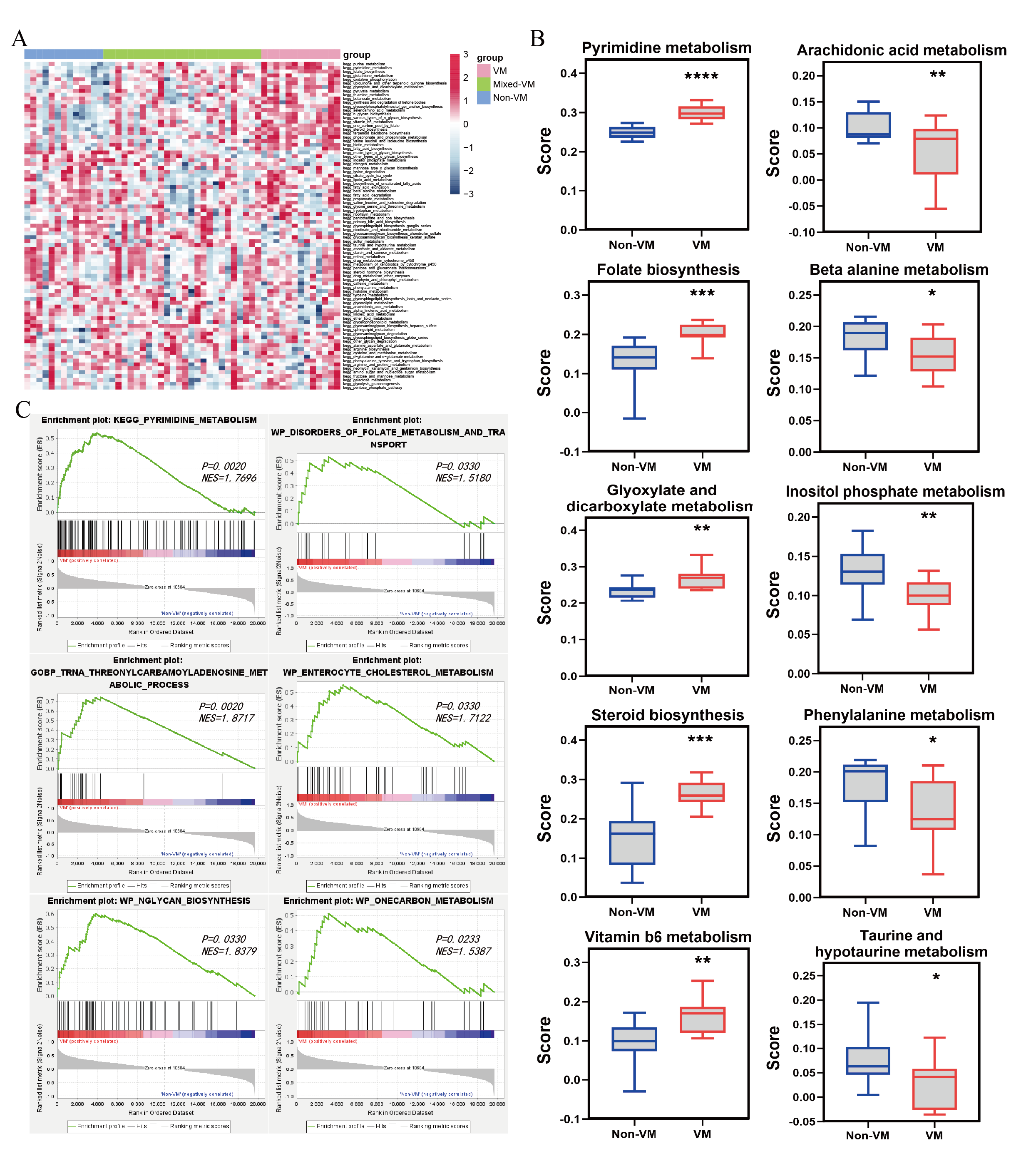


**Figure S4** VM subtypes exhibit distinct metabolic reprogramming patterns. **(A)** Heatmap displaying the differential metabolic pathway activities across the three VM subtypes based on ssGSEA scores of metabolic pathway gene sets. **(B)** Box plots comparing the enrichment scores of specific metabolic pathway gene sets between VM and Non-VM subtypes. * indicates q < 0.05; ** indicates q < 0.01; *** indicates q < 0.001; **** indicates q < 0.0001. **(C)** Gene Set Enrichment Analysis (GSEA) revealing significantly activated metabolic pathways in VM subtypes.


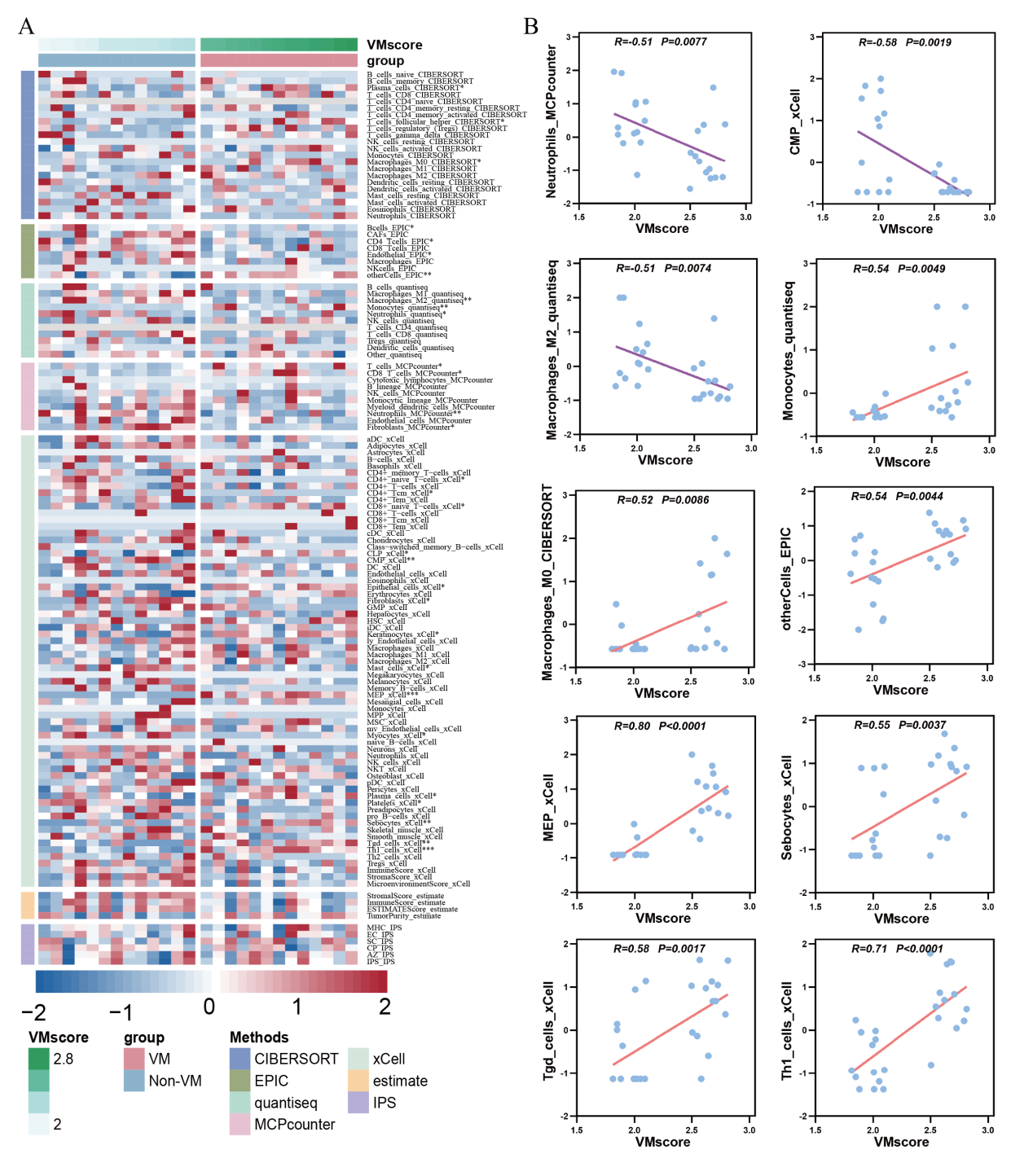


**Figure S5** Association between VM subtypes and tumor immune microenvironment composition in bladder cancer. **(A)**Heatmap showing the relative enrichment scores of immune and stromal cell types in VM and Non-VM subtypes, calculated using seven immune infiltration estimation algorithms. **(B)** Pearson correlation analysis revealing relationships between VM scores and various immune/stromal cell types. Red lines indicate positive correlations, while purple lines indicate negative correlations; all displayed correlations are statistically significant *(P < 0.05*).


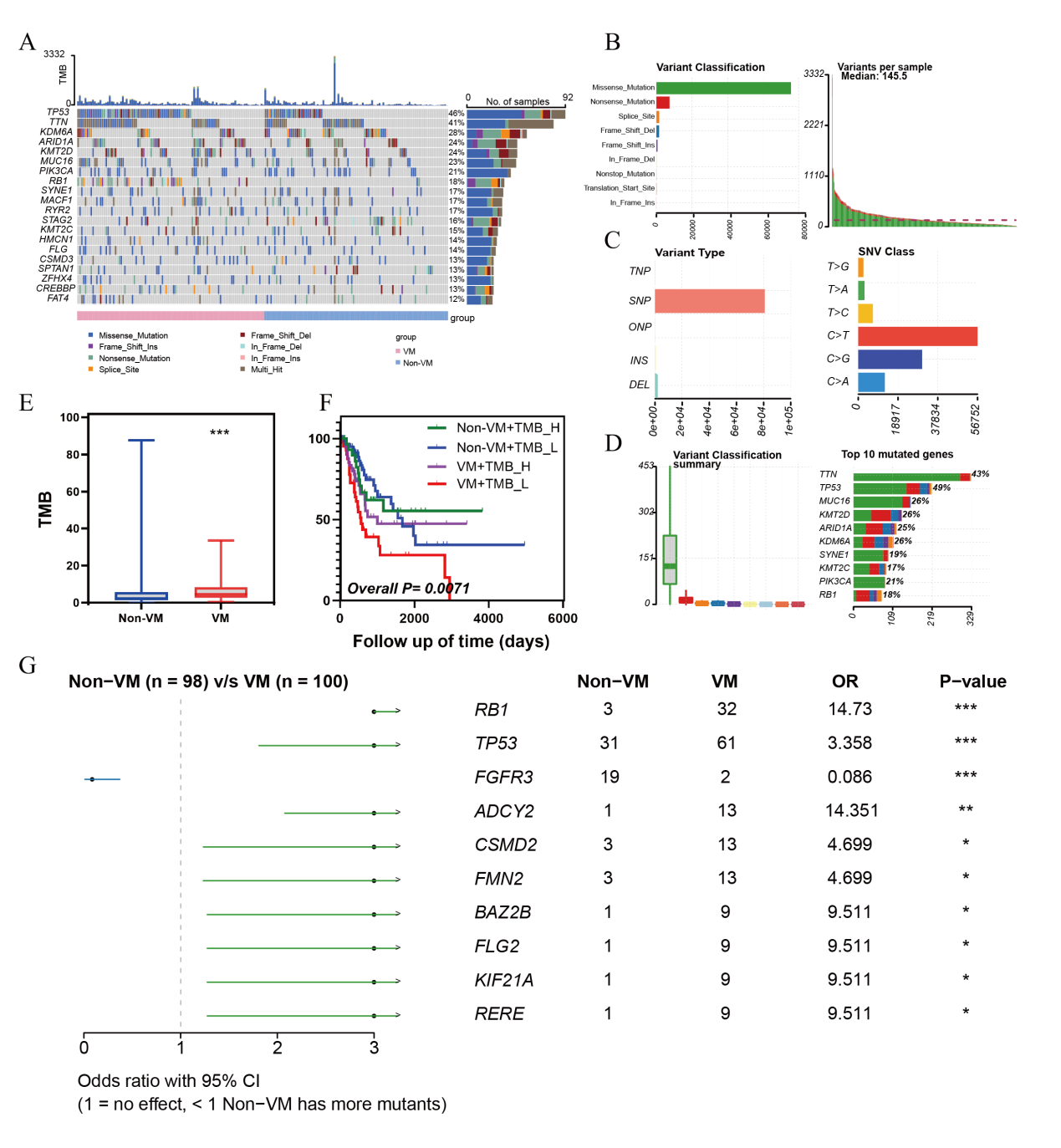


**Figure S6** Mutational landscape and tumor neoantigen characteristics of VM subtypes in MIBC. **(A)** Waterfall plot displaying differences in somatic mutation profiles between VM and Non-VM subtypes. **(B-D)** Summary of somatic mutations in the TCGA-BLCA cohort: (B) distribution of variant classifications and total mutation counts per sample; (C) variant types and single nucleotide variant (SNV) classes; (D) most frequently mutated genes and their mutation type distribution. **(E)**Comparison of tumor mutation burden (TMB) between VM and Non-VM subtypes. * indicates q < 0.05; ** indicates q < 0.01; *** indicates q < 0.001. **(F)** Kaplan-Meier curves for overall survival of patients stratified by VM subtype and TMB status (TMB_H: high TMB group; TMB_L: low TMB group). **(G)**Forest plot of differentially mutated genes between VM and Non-VM subtypes.


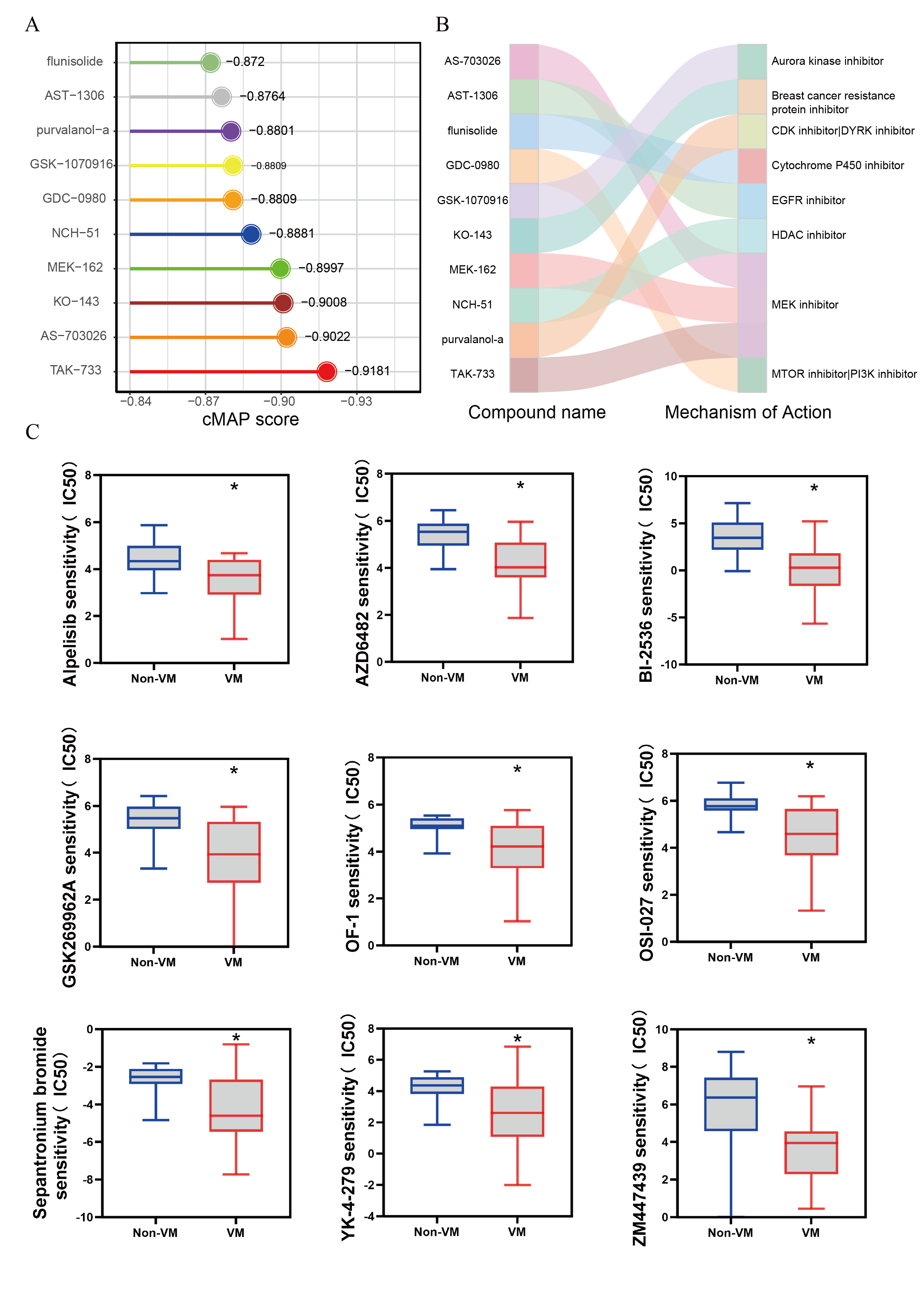


**Figure S7** Drug sensitivity analysis of VM subtypes. **(A)** Bar plot showing the top 10 drugs most significantly negatively correlated with the VM transcriptional signature. **(B)** Sankey diagram illustrating the association between the 10 candidate drugs in (A) and their known mechanisms of action. **(C)**Box plots demonstrating significantly lower half-maximal inhibitory concentrations (IC_50_) for VM subtypes compared to Non-VM subtypes across nine compounds: Alpelisib, AZD6482, BI-2536, GSK269962A, OF-1, OSI-027, Sepantronium bromide, YK-4-279, and ZM447439.* indicates q < 0.05.


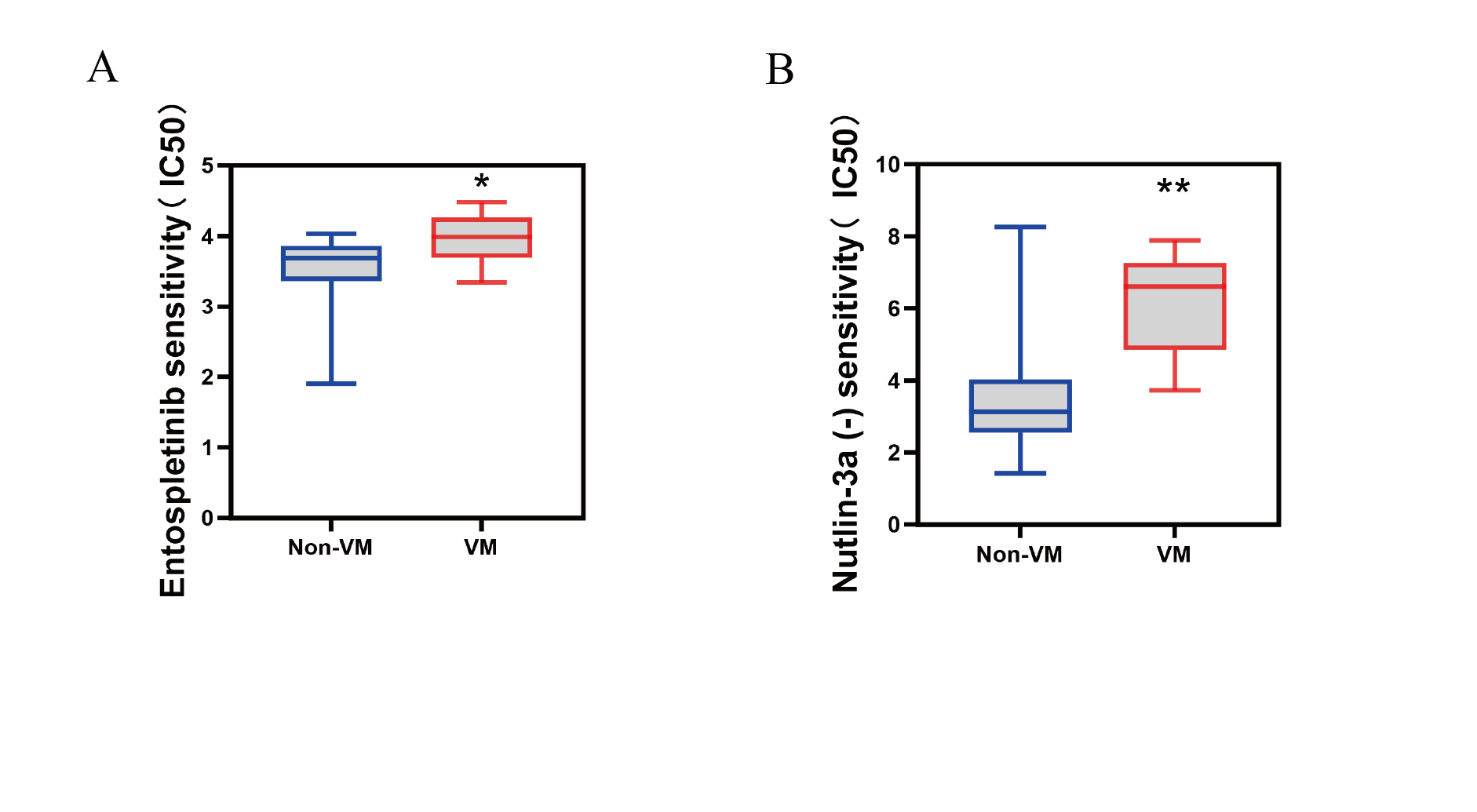


**Figure S8** The VM subtype of bladder cancer is associated with resistance to Entospletinib and Nutlin-3a (-). **(A-B)** Box plots comparing the half-maximal inhibitory concentration (IC50) of Entospletinib and Nutlin-3a (-) between VM subtype and non-VM subtype. The VM subtype exhibits significantly higher IC50 values. *indicates q < 0.05; ** indicates q < 0.01.


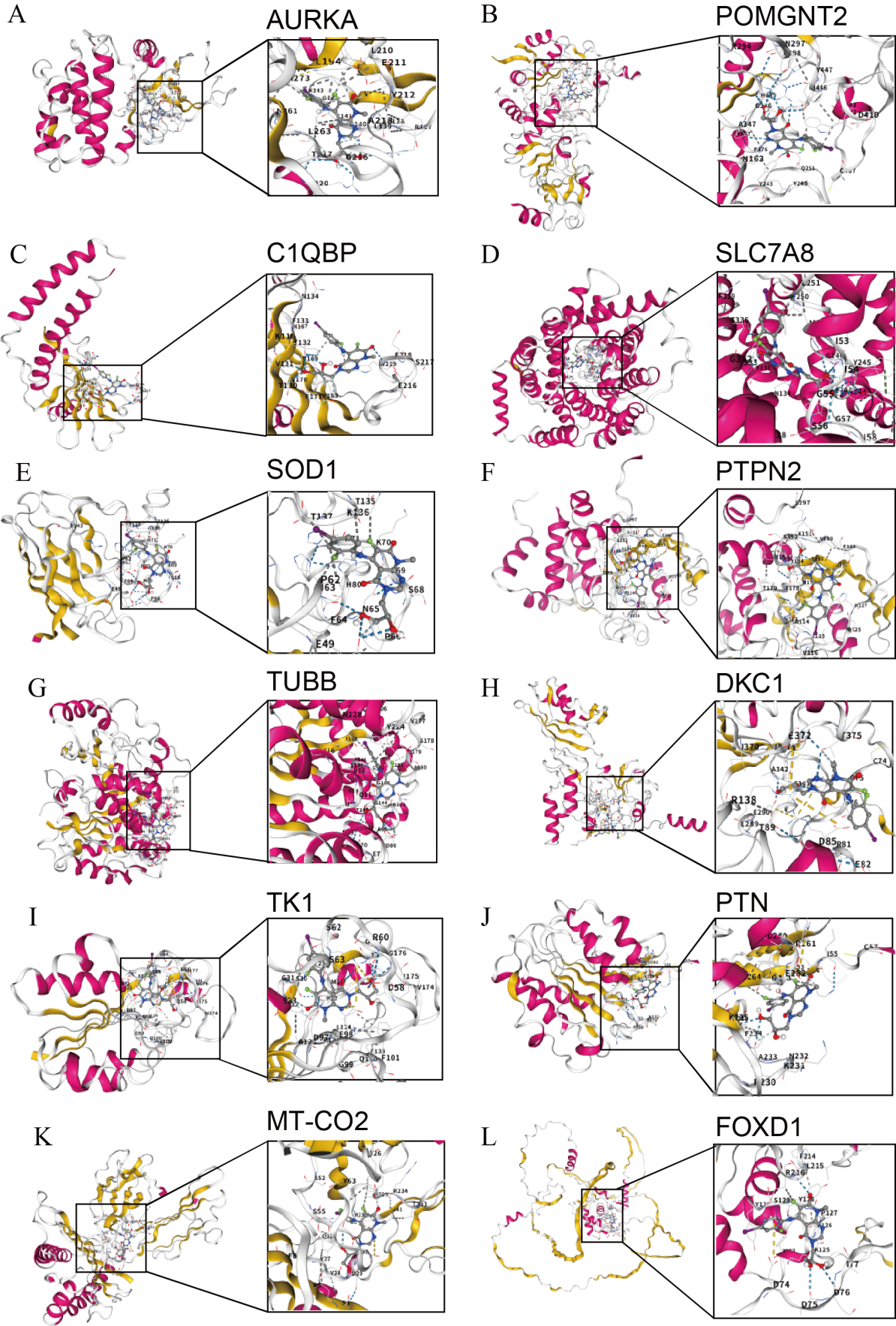


**Figure S9** Analysis of docking modes between core target proteins and the ligand molecule. **(A-L)**Three-dimensional structural diagrams of molecular docking showing the interactions between TAK-733 and 12 core target proteins (AURKA, POMGNT2, C1QBP, SLC7A8, SOD1, PTPN2, TUBB, DKC1, TK1, PTN, MT-CO2, and FOXD1), respectively.


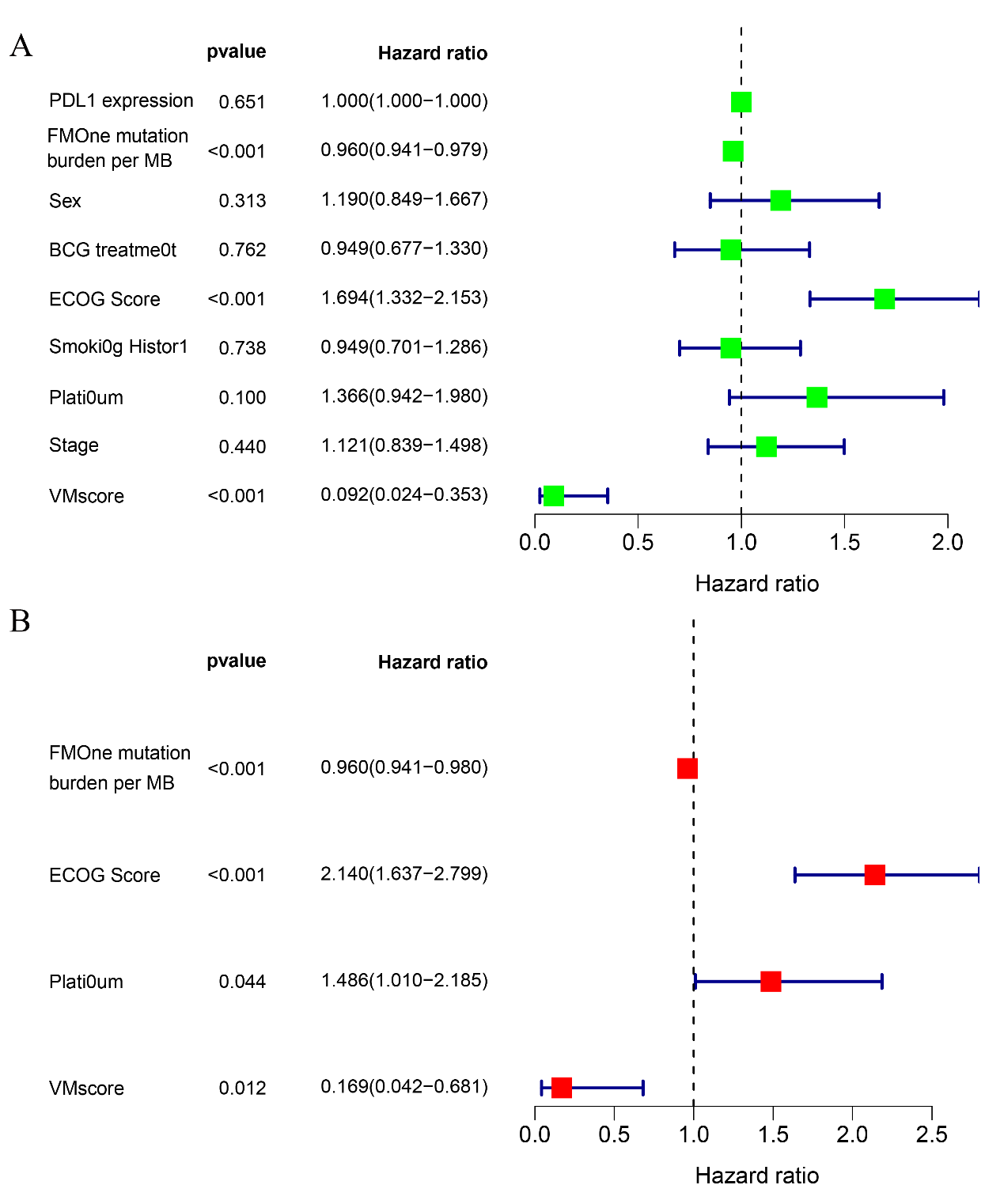


**Figure S10** Univariate and multivariate Cox regression analyses assessing the prognostic value of the VM score in the IMvigor210 immunotherapy cohort.**(A)** Forest plot showing the results of univariate Cox regression analysis for overall survival.**(B)** Forest plot showing the results of multivariate Cox regression analysis for overall survival.
